# Automated PACS-integrated pipeline for TractSeg-based segmentation of the arcuate fasciculus in patients with hearing loss

**DOI:** 10.3389/fnins.2026.1830648

**Published:** 2026-05-19

**Authors:** Sara Kierońska-Siwak, Beata Zwierko, Hanna Mackiewicz-Nartowicz, Agata Kozakiewicz-Rutkowska, Anna Sinkiewicz, Alina Jaroch, Marietta Bracha, Grzegorz Meder, Dariusz Grzanka

**Affiliations:** 1 Department of Clinical Pathomorphology, Faculty of Medicine, Collegium Medicum in Bydgoszcz, Nicolaus Copernicus University in Torun, Bydgoszcz, Poland; 2 Department of Neurosurgery, Stereotactic and Functional Neurosurgery, Jan Biziel Hospital No. 2 in Bydgoszcz, Bydgoszcz, Poland; 3 Department of Electroradiology, Faculty of Health Sciences, Collegium Medicum in Bydgoszcz, Nicolaus Copernicus University in Torun, Bydgoszcz, Poland; 4 Department of Otolaryngology, Phoniatry and Audiology, Collegium Medicum in Bydgoszcz Nicolaus Copernicus University in Torun, Bydgoszcz, Poland; 5 Department of Geriatrics, Faculty of Health Sciences, Collegium Medicum in Bydgoszcz, Nicolaus Copernicus University in Torun, Bydgoszcz, Poland; 6 Department of Interventional Radiology, Jan Biziel University Hospital No. 2, Bydgoszcz, Poland

**Keywords:** arcuate fasciculus, automated pipeline, diffusion MRI, tractography, TractSeg

## Abstract

**Objective:**

While tractography is used to determine the anatomical course of white matter tracts, it can often be imprecise and time-consuming, which can be a problem when comparing large groups of patients. The aim of this study is to compare the automated process of arcuate fasciculus determination using the TractSeg algorithm with manual AF determination in DSI studio software.

**Methods:**

The process of importing the structured MPRAGE sequence and raw diffusion-weighted images from the PACS system, performing the TractSeg algorithm, superimposing the bilateral AFs obtained on the MPRAGE image and exporting this composite image to the PACS system was automated. This procedure was used to segment the arcuate fasciculus in 25 patients with hearing loss.

**Results:**

The automated algorithm was able to delineate the arcuate fasciculus bilaterally in all 25 patients, while the manual reference method and automated tractography based on DSI Studio software failed in three and one patient, respectively. TractSeg showed a mean distance of 2.0 ± 0.7 mm from manual segmentation, compared with 2.8 ± 1.0 mm for DSI Studio Auto-Tracking. In addition, TractSeg appeared to involve larger portions of the medial AF fibres than the other methods.

**Conclusion:**

The TractSeg algorithm has shown high efficacy in segmenting the arcuate fasciculus in patients with hearing loss. The algorithm is fast to run and has great potential to optimise and improve neural pathway delineation.

## 1 Introduction

In patients with hearing disorders, especially those of a sensorineural nature, the assessment of cortical and subcortical structures responsible for auditory stimulus processing and language integration is becoming increasingly important. One of the key white matter pathways involved in these processes is the arcuate fasciculus, which connects the auditory cortex of the temporal lobe to the lingual areas of the frontal lobe. Abnormalities in its structure, organisation or continuity can affect hearing development and function, particularly in the context of central auditory processing disorders, congenital or acquired hearing loss, and abnormal speech development (Dick and Tremblay, 2012; Ivanova et al., 2021; Gonçalves and Monteiro, 2023; Ostertag et al., 2023).

In recent years, diffusion imaging techniques such as diffusion tensor imaging (DTI) and white matter tractography (WMT) have made it possible to non-invasively assess the integrity of the arcuate bundle at both the microstructural and topographic levels. These methods are recognised as optimal tools for analysing the course and organisation of white matter pathways, a point that has also been repeatedly emphasised in the context of neurosurgery and surgery planning, where tractography provides important support in assessing the relationship of deep structures to lesions (Soni et al., 2017; Kieronska and Słoniewski, 2020; Di Cristofori et al., 2021; Zoli et al., 2021).

Identifying the location and morphology of the arcuate bundle in people with hearing loss can provide valuable information about the neuroanatomical basis of auditory disorders, as well as enabling a better understanding of compensatory brain reorganisation (Abhinav et al., 2014; Pasquini et al., 2022). In addition, the integration of tractography may potentially support clinical applications when robust and reproducible automated workflows become available.

Despite the growing number of applications, traditional tractography requires precise placement of initiation points, manual optimisation of parameters and time-consuming verification of fibre runs by experienced specialists. This process, as indicated in the literature, is prone to significant inter-operator variation and can be a barrier to routine clinical use. Consequently, the development of semi-automatic and fully automated methods that provide reproducible and rapid results seems crucial for the continued use of tractography in the assessment of hearing disorders (Wasserthal et al., 2018; Chamberland et al., 2021; Aksoy et al., 2023).

### 1.1 The role of tractography in the assessment of central auditory pathways

In patients with hearing loss, it is important not only to determine the extent of damage to the peripheral auditory system, but also to understand how reduced acoustic input affects the development or degeneration of central white matter pathways. The arcuate bundle is one of the main connections of the language network and is involved in the perceptual-expressive integration of speech, phonological memory and processes related to language learning. Changes in its structure have been described in people with auditory processing disorders, prelingual deafness, aphasia or language learning difficulties, among others (Husain et al., 2011; Ivanova et al., 2021).

DTI together with tractography allows quantitative assessment of parameters such as fractional anisotropy (FA), radial diffusion (RD) or pathway volume, which can be biomarkers of neuronal plasticity or degeneration. Previous studies suggest that a lack of adequate auditory stimulation can lead to functional reorganisation and morphometric changes within the arcuate fasciculus, further emphasising the importance of its assessment in the hearing loss patient population (Farquharson et al., 2013; de Lange et al., 2016).

### 1.2 Available tractography methods

There are a number of methods to reconstruct white matter pathways. In clinical practice, mainly deterministic and probabilistic tractography models based on DTI data are used. However, their use is limited by a number of factors: high dependence on operator experience, the need for time-consuming ROI setting, variability of results depending on the algorithm and reconstruction parameters, susceptibility to errors in fibre crossing areas (Campbell and Pike, 2014; Jeurissen et al., 2014; Schilling et al., 2019).

As also highlighted in studies on tractography in neurosurgery, even using the same software parameters, differences between operators can lead to significant variability in results. As a result, tractography is not routinely used in the assessment of patients with hearing loss, even though it could provide significant diagnostic value (Jeurissen et al., 2014).

Automatic methods, including direct segmentation algorithms (e.g., models using convolutional networks), have also been developed in recent years and can significantly simplify and speed up the analysis process (Wasserthal et al., 2019; Wang et al., 2021; Li et al., 2024). They enable more reproducible identification of white matter pathways, reducing the impact of operator error and increasing potential clinical utility (Zhang et al., 2018).

The aim of the present study was methodological rather than clinical. Patients with hearing loss were included to evaluate the robustness and clinical workflow integration of a fully automated TractSeg-based segmentation pipeline in a cohort representing non-normal white matter anatomy, rather than to investigate neurobiological mechanisms of hearing impairment.

## 2 Materials and methods

### 2.1 Patients information

The study included 25 patients with sensorineural hearing loss following COVID-19 infection (11 men, 14 women), aged 39 to 74 years (mean 52.9 ± 10.1 years; median 49 years). All patients underwent audiometric testing, which determined mean hearing thresholds expressed in dB HL for the right and left ears separately. Mild to moderate hearing loss predominated in the analysed group, with some patients experiencing higher hearing thresholds exceeding 40–60 dB HL (Table 1). In most cases, hearing loss was bilateral, although some patients demonstrated asymmetry in hearing thresholds between the ears. The variation in the degree and nature of hearing loss indicates potential heterogeneity of changes within the central auditory pathways, which was an important justification for the assessment of white matter structures, in particular the arcuate fasciculus, using diffusion imaging and tractography methods.

**Table 1:** Demographic data patients.

Variable	Value
Number of patients	25
Age (years), mean ± SD	52.9 ± 10.1
Age (years), median (range)	49 (39–74)
Sex	11 male / 14 female
Type of hearing loss	Sensorineural
Hearing thresholds – right ear (dB HL)	13–68 dB HL
Hearing thresholds – left ear (dB HL)	9–49 dB HL
Degree of hearing loss	Predominantly mild to moderate
Bilateral hearing loss	Predominant in the cohort
Asymmetric hearing thresholds	Present in a subset of patients
MRI scanner	Philips Ingenia 3.0 T
Diffusion acquisition	64 directions, b = 1,000 s/mm^2^

### 2.2 Data acquisition

The examination was performed using a 3.0 T MRI system (Ingenia, Philips) with a 32-channel head coil, according to a protocol designed to assess hearing impairment in post-COVID-19 patients. The protocol included 3D T1 TFE imaging (FOV 250 × 250 × 181 mm, voxel size 1 × 1 × 1 mm, TE/TR 3.8/8.3 ms, acquisition time 6:01), 3D FLAIR (FOV 240 × 240 × 180 mm, voxel size 1 × 1 × 1 mm, TE/TR 318/4800 ms, time 5:22), and 3D T2 imaging (TE/TR 259/2500 ms, time 4:13), complemented by SWI (voxel size 0.65 × 0.65 × 2 mm, TE/TR 7.2/31 ms, time 3:52), DTI with 64 diffusion directions (voxel size 2 × 2 × 2 mm, TE/TR 87/8835 ms, time 12:03), b-value 1,000 s/mm^2^, 3D pCASL perfusion imaging, and resting-state fMRI (voxel size 3.1 × 3.1 × 3.1 mm, TE/TR 30/2500 ms, time 10:40). Susceptibility-induced geometric distortions were corrected using a field map–based approach acquired during the same imaging session. This comprehensive protocol enabled simultaneous assessment of structural brain changes, microhemorrhages, integrity of neural pathways, cerebral perfusion, and functional connectivity.

### 2.3 Automatic segmentation of the arcuate fasciculus

In order to automate the arcuate fasciculus reconstruction process, a complete pipeline was developed including data import, preprocessing, performing automated segmentation and then preparing the results in clinical format. Raw diffusion-weighted imaging (DWI) and T1-weighted sequence images were acquired from the PACS and stored on a work server, where a specially developed script monitored the influx of new data. Once a DICOM series was detected, the script analysed the file headers to identify T1 sequences and diffusion sets and initiated an automatic conversion procedure to NIfTI format using the dcm2niix tool, which also generated the corresponding b-values and b-vectors files.

In the next step, the diffusion data were subjected to correction of distortions due to patient movement and magnetic field gradients, using dedicated eddy currents correction tools.

Automated segmentation of the arcuate fasciculus was performed using TractSeg v2.9, a deep learning–based direct white matter bundle segmentation framework (Wasserthal et al., 2019). The method uses convolutional neural networks trained on diffusion-derived fibre orientation information to predict bundle-specific masks directly in voxel space, without the need for manual ROI placement or streamline tracking (Wasserthal et al., 2018, 2019). Diffusion data served as input to the model, which generated bilateral arcuate fasciculus segmentation masks. The resulting probability maps were thresholded to obtain binary representations of the tract and subsequently registered to the T1-weighted image for anatomical visualisation and further quantitative analysis.

To ensure the spatial correspondence of the bundle mask with the anatomical images, the segmentations were subjected to a two-stage registration:

1. first to the native space of the corrected diffusion images,
2. then to the T1 sequence using linear transformations to compensate for any differences in head positioning between imaging sessions.

After registration, the bundle mask was thresholded (typically at 0.8) to obtain a binary representation of the most likely part of the pathway. The segmentation prepared in this way was superimposed on the T1-weighted image, overwriting the intensity values in the areas corresponding to the fibres so that the segmentation was clearly visible in standard radiological viewers.

At the end of the pipeline, it generated a series of DICOM files containing a T1 image with superimposed bundle-branch segmentation, retaining the original study metadata but with a new series identifier. These files were automatically sent back to the PACS, where they could be assessed by a radiologist or clinical specialist without the need for additional software. The entire process was designed to require minimal operator intervention and to produce a finished segmentation within minutes of completing the data acquisition.

### 2.4 Automatic tractography in DSI studio

For comparison with the main automatic segmentation method, the *Auto-Tracking* function available in DSI Studio software 2025.04 was used to automatically reconstruct selected bundles of white matter based on atlases and built-in anatomical rules. The raw diffusion data was first imported into DSI Studio and reconstructed using Generalised Q-Sampling Imaging (GQI), which has a high robustness to the noise and complex fibre patterns typical of the junctional area running through the temporal–parietal region. The value of the discrete sampling ratio was set to a typical value of 1.25 for this model.

After reconstruction, the pathway corresponding to the arching bundle (AF) was selected from the atlas list. The tracking process used standard automatic parameters: The Auto-Tracking procedure was performed using standard deterministic tractography parameters: angular threshold 60°, minimum fiber length 20 mm, maximum length 300 mm, and topology-informed pruning (TIP) enabled. These settings were selected to balance anatomical plausibility and reproducibility and were consistent across all subjects. The autotrack tolerance has been set to a default value, allowing for slight deviations from the atlas model of the route. The resulting fibres were exported and superimposed on the T1-structured image to allow direct comparison with automated and manual segmentations.

**Figure 1:**
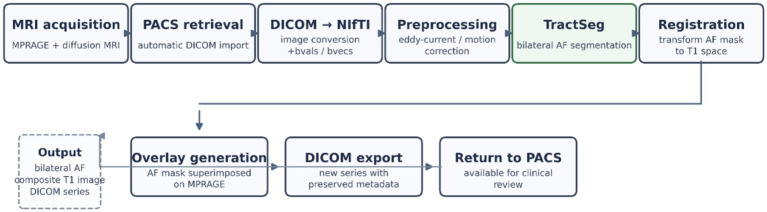
Automated PACS-integrated TractSeg pipeline for arcuate fasciculus segmentation.

The overall workflow of the automated PACS-integrated segmentation pipeline is illustrated in Figure 1.

### 2.5 Manual segmentation in DSI studio

Manual segmentation of the arcuate fasciculus was carried out in the DSI Studio as a reference method, analogous to the procedure described in the article, in which manual reconstruction of pyramidal tracts served as a comparative standard. Manual tractography was performed by an experienced operator. Manual segmentation was performed without access to automated segmentation results.

After importing the diffusion data, GQI reconstruction was performed with a sampling ratio of 1.25, due to its high performance in areas of complex fibre architecture, characteristic of the AF region.

The operator then manually delineated regions of interest (ROIs) according to the anatomical course of the arcuate bundle. ROIs included:

1. Temporal region (posterior part of the superior temporal lobe)—as the initial area,
2. Parietal–temporal region (over the junction of AF and SLF fibres)—as a checkpoint to limit erroneous streams,
3. Frontal region (around the inferior frontal gyrus)—as the terminal area of the bundle.

Deterministic tractography was performed using QA as the tracking parameter with an angular threshold of 60°, minimum length 20 mm, and maximum length 300 mm and the generation of up to 1,000,000 seeding samples. Once a complete set of fibres had been produced, the operator removed all streams with a course inconsistent with the anatomy of the arcuate bundle (e.g., fibres crossing into the parietal lobe, running too medially or towards highly crossed structures).

The manual segmentation, performed by an experienced operator, provided a benchmark for comparison with the automatic methods, allowing an assessment of their compatibility and ability to correctly represent the AF course.

**Figure 2:**
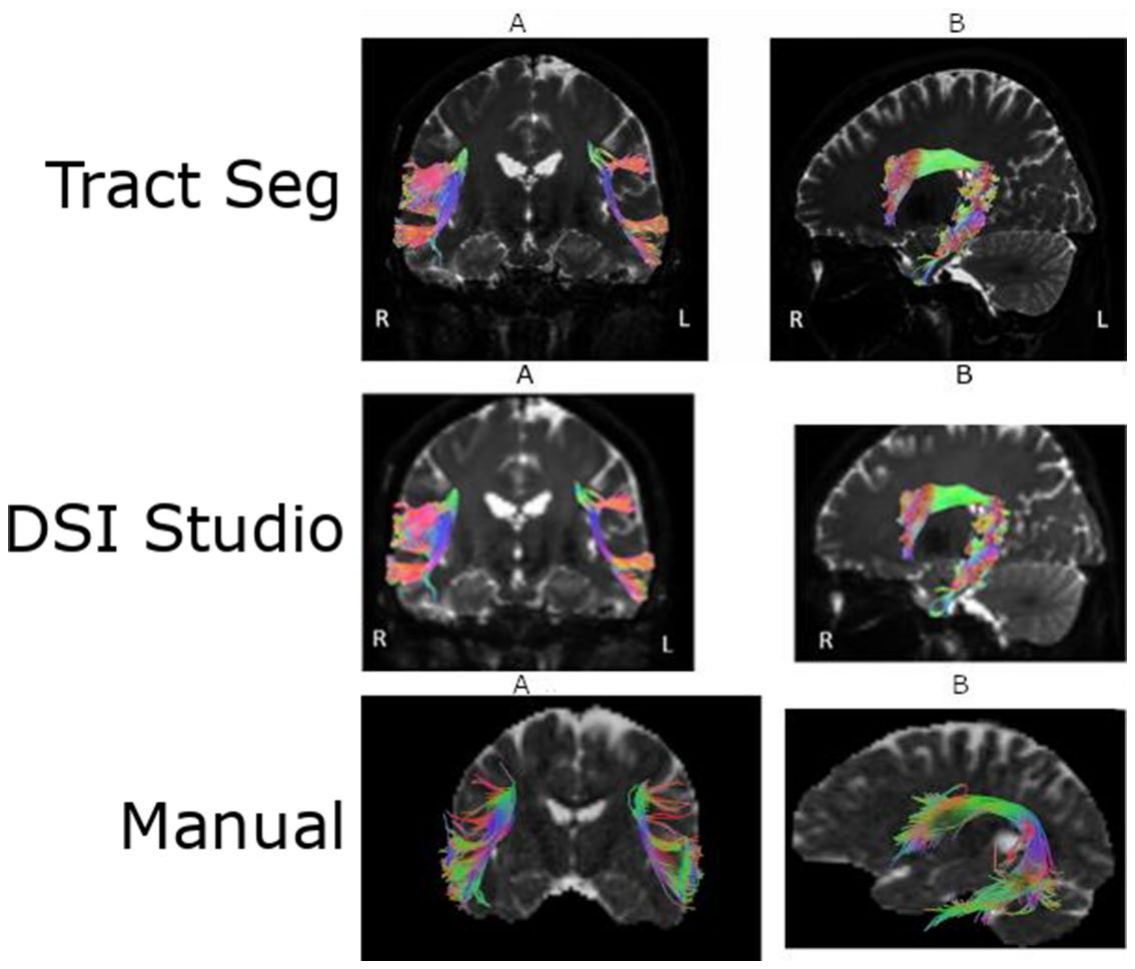
Comparison of arcuate fasciculus reconstructions obtained using different tractography approaches. The first row shows automated segmentation performed with the TractSeg algorithm, the second row presents automatic tractography generated with DSI Studio Auto-Tracking, and the third row demonstrates manual tractography performed in DSI Studio using anatomically defined regions of interest. Coronal views are shown in the left column **(A)**, whereas sagittal views are presented in the right column **(B)**.

Representative examples of arcuate fasciculus reconstruction obtained using the different tractography approaches are shown in Figure 2.

### 2.6 Quantitative comparison

To assess spatial agreement between automated and manual segmentation of the arcuate fasciculus, a distance-based analysis was performed. All tract masks were resampled to the same voxel grid in T1-weighted image space using nearest-neighbour interpolation.

For each voxel belonging to the manually segmented arcuate fasciculus, the Euclidean distance to the nearest voxel in the corresponding automated segmentation was calculated, separately for TractSeg and DSI Studio Auto-Tracking. Distances were summarised for each hemisphere using the mean and the 95th percentile. Group-level results are reported as mean ± standard deviation across subjects.

Distance-based metrics were preferred over overlap-based measures, as automated methods—particularly TractSeg—tended to produce larger segmentations, which could bias overlap coefficients despite anatomically plausible tract representations.

### 2.7 Statistical analysis

Statistical analysis was performed to compare the spatial correspondence between automated arcuate fasciculus segmentation methods (TractSeg and Auto-Tracking in DSI Studio) and the reference method based on manual tractography. For each cerebral hemisphere, the Euclidean distance between voxels belonging to the manually segmented arcuate fasciculus and the nearest voxels in the corresponding automated segmentation was calculated. Normality of the obtained data distribution was assessed using the Shapiro–Wilk test. When assumptions of normality were met, the Student’s t-test for paired samples was used to compare methods; when these assumptions were not met, the nonparametric Wilcoxon signed-rank test was used. Results are presented as means ± standard deviation and 95th percentile distances. The level of statistical significance was set at *p* < 0.05. Analyses were performed separately for the left and right cerebral hemispheres.

## 3 Results

### 3.1 Tractography results

The TractSeg algorithm was able to delineate the arcuate fasciculus bilaterally in all 25 patients, while the manual reference method and automatic tractography based on DSI Studio software failed in three and one patient, respectively. In all cases, the TractSeg segmentations were very close to the manual segmentations. In addition, TractSeg appeared to involve larger portions of the medial arcuate fasciculus fibres than the other methods.

**Figure 3:**
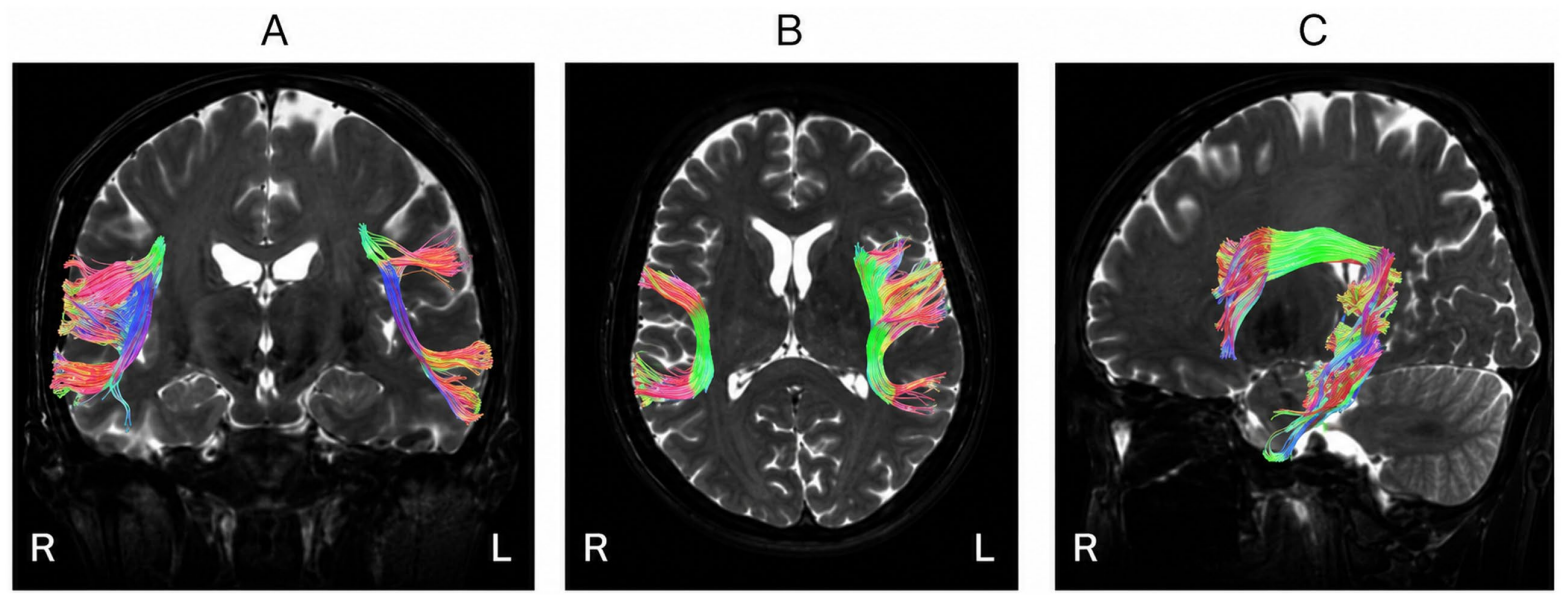
Representative anatomical views of the arcuate fasciculus. **(A)** Coronal view showing bilateral arcuate fasciculus fibres in the temporo-parietal region. **(B)** Axial view illustrating the lateral distribution of the tract along the white matter of both hemispheres. **(C)** Sagittal view demonstrating the characteristic arcuate trajectory connecting posterior temporal regions with the inferior frontal gyrus.

Representative anatomical visualisations of the arcuate fasciculus obtained from diffusion tractography are presented in Figure 3.

### 3.2 Quantitative comparison

A quantitative comparison between the automated segmentations and the manual method showed very good spatial agreement in most lengths of the arcuate fasciculus, the best in its middle segments. Quantitative analysis demonstrated a high degree of spatial agreement between automated segmentations and the manual reference method.

Distances were computed voxel-wise in T1 space as the Euclidean distance from each manual AF voxel to the nearest voxel in the automated segmentation and summarised per subject as mean and 95th percentile. Group results are reported as mean ± SD across subjects. TractSeg showed a mean distance of 2.0 ± 0.7 mm (95th percentile 5.0 mm), whereas DSI Studio Auto-Tracking showed larger deviations (2.8 ± 1.0 mm; 95th percentile 7.0 mm; Table 2).

**Table 2:** Spatial agreement between manual and automated arcuate fasciculus segmentations (distance in mm).

Hemisphere	Method	Mean distance (mm)	95th percentile (mm)
Left	TractSeg	2.0 ± 0.7	5.0
Left	DSI Studio Auto	2.8 ± 1.0	7.0
Right	TractSeg	2.1 ± 0.8	5.2
Right	DSI Studio Auto	2.9 ± 1.1	7.2

The smallest distances were observed in the mid-portions of the arcuate fasciculus, whereas larger discrepancies occurred near cortical terminations and in regions of complex fiber architecture. In these areas, TractSeg segmentations remained closer to the manual reference than DSI Studio–based automated tractography. Quantitative analysis was performed in subjects with successful manual reconstruction (*n* = 22); cases with failed manual segmentation were excluded.

## 4 Discussion

In the present study, the TractSeg algorithm was shown to enable bilateral segmentation of the arcuate fasciculus in all hearing-impaired patients studied, outperforming both manual reference tractography and automated tractography based on DSI Studio software. Distance-based quantitative analysis supported the qualitative observations, demonstrating closer agreement of TractSeg with the manual reference compared to DSI Studio–based automated tractography, particularly in the mid-portions of the arcuate fasciculus (Campbell and Pike, 2014; Zhang et al., 2018; Gruen et al., 2025).

The proposed pipeline demonstrates how automated tractography can be integrated directly into routine clinical PACS workflows.

These results are consistent with previous reports showing the high reliability of TractSeg in segmenting major white matter pathways, even in clinical populations characterised by altered anatomy or reduced diffusion signal quality (Wasserthal et al., 2018; Gruen et al., 2025).

The segmentations obtained with the TractSeg algorithm showed very good spatial agreement with the manual segmentation, especially in the middle segments of the arcuate fasciculus. The differences observed were mainly in marginal areas and regions with complex fibre architecture, a phenomenon typical of tractography based on diffusion data and has been repeatedly reported in the literature. It should be emphasised that the lack of an unambiguous “ground truth” in white matter pathway imaging limits the ability to unequivocally assess the absolute accuracy of each method (Maier-Hein et al., 2017; Schilling et al., 2019).

Compared to the other methods, TractSeg appeared to involve larger sections of the medial fibres of the arcuate fasciculus (Wasserthal et al., 2019; Zhang et al., 2022). This phenomenon may be due to the characteristics of the algorithm based on direct deep-learning segmentation which is not constrained by the stream-tracking rules and anisotropy thresholds used in classical tractography. Similar observations were also reported for other white matter pathways, where TractSeg showed increased sensitivity while maintaining an anatomically plausible pathway (Schilling et al., 2019).

A significant advantage of the proposed approach is the full automation of the segmentation process and the integration of the results with the PACS, which enables the use of tractography without the need for dedicated software and without involving an experienced operator. In the context of the increasing workload in clinical radiology, such solutions could increase the availability of tractography analyses and facilitate their potential use in the diagnosis and management planning of patients with hearing impairment (Gramegna et al., 2025).

### 4.1 The research limitations

This study has several limitations. Firstly, there is no objective reference standard that allows for an unambiguous assessment of the accuracy of arcuate fasciculus segmentation. Secondly, the analysis was performed on data from one centre and one imaging protocol, which may limit the generalisability of the results. Nevertheless, given previous reports confirming the robustness of the TractSeg algorithm to input variability, the results obtained indicate its potential usefulness in a wider clinical context (Maier-Hein et al., 2017; Schilling et al., 2019).

## Data Availability

The raw data supporting the conclusions of this article will be made available by the authors, without undue reservation.
